# Reporting of critical care trial abstracts: a comparison before and after the announcement of CONSORT guideline for abstracts

**DOI:** 10.1186/s13063-017-1786-x

**Published:** 2017-01-21

**Authors:** Akira Kuriyama, Naomi Takahashi, Takeo Nakayama

**Affiliations:** Department of Health Informatics, Kyoto University School of Public Health, Yoshida-Konoe-cho Sakyo-ku, Kyoto, 606-8501 Japan

**Keywords:** Randomized controlled trials, CONSORT for abstracts, Quality of reports, Abstracts, Adherence, Systematic review

## Abstract

**Background:**

An extension of the Consolidated Standards of Reporting Trials (CONSORT) statement provides a checklist of items to improve the reporting quality of abstracts of randomized controlled trials (RCTs). However, authors of abstracts in some fields have poorly adhered to this guideline. We did an extensive literature survey to examine the quality of reporting trial abstracts in major critical care journals before and after announcement of the CONSORT guideline for abstracts.

**Methods:**

We reviewed abstracts of RCTs published in four major critical care journals with publication dates ranging from 2006 to 2007 (pre-CONSORT) and from 2011 to 2012 (post-CONSORT): *Intensive Care Medicine* (ICM), *Critical Care* (CC), *American Journal of Respiratory and Critical Care Medicine* (AJRCCM), and *Critical Care Medicine* (CCM). For each item in the CONSORT guideline for abstracts, we considered that an abstract was well-reported when it reported a relevant item and adhered to the guideline. Our primary outcomes were to describe the proportion of abstracts that adhered to the guideline for each item in each period and the changes between the two periods. Pearson’s chi-square analysis was performed to compare adherence to the guideline between the two periods.

**Results:**

Our inclusion criteria yielded 185 and 166 abstracts from pre- and post-CONSORT periods, respectively. Less than 50% of abstracts adequately reported trial design (16.3%), participants (44.0%), outcomes in methods (49.4%), randomization (1.8%), blinding (4.2%), numbers randomized (37.4%) and analyzed (8.4%), recruitment (4.2%), outcomes in results (16.9%), harms (27.7%), trial registration (42.2%), and funding (13.9%) in the recent period. There was significant improvement in reporting title, primary outcomes in both methods and results, interventions, harms, trial registration, and funding between the two periods (*p* < 0.05). Improvements were seen in reporting of participants in the Methods sections in CCM, as well as in outcomes in results and trial registration in AJRCCM and CCM, between the two periods. A significant decline was noted in reporting of interventions in Methods sections in AJRCCM and ICM, as well as the numbers randomized in Results sections in CC, over time.

**Conclusions:**

Reporting of some items in abstracts for critical care trials improved over time, but the adherence to the CONSORT guideline for abstracts was still suboptimal.

## Background

Randomized controlled trials (RCTs) are studies designed to compare therapeutic or preventive interventions in medicine. Clear, transparent, and sufficient abstracts are required when reporting RCTs, because readers consider articles reporting RCTs according to the information provided in abstracts. In particular, health professionals with limited access to the full texts rely on abstracts, which might eventually influence healthcare decisions [1, 2]. Abstracts of conference proceedings are also important resources in conducting systematic reviews, which would otherwise introduce a form of bias if excluded [3].

An extension of the Consolidated Standards of Reporting Trials (CONSORT) published in 2008 provides a checklist of items to be included in journal or conference abstracts reporting RCTs [4]. Overall, studies evaluating the reporting quality of trial abstracts in other fields have shown poor adherence to the CONSORT guideline for abstracts since publication of the guideline [5–14]. Some studies have also reported significant improvements in adherence, but still suboptimal adherence to the guideline, comparing the situation before and after publication of this guideline [15–17]. To date, little is known about adherence to the CONSORT guideline for abstracts or changes in the reporting of trial abstracts from before to after the publication of this guideline in the domain of critical care. In this study, we examined the quality of reporting trial abstracts in major critical care journals before and after announcement of the CONSORT guideline for abstracts.

## Methods

### Study selection

We conducted a search of PubMed to identify all RCTs published in major critical care journals before and after the release of the CONSORT guideline for abstracts in 2008. We included critical care journals that focused on general critical care topics, and we excluded those on subspecialty or specific areas. As of 2006 and 2007, *American Journal of Respiratory and Critical Care Medicine* (AJRCCM), *Intensive Care Medicine* (ICM), *Critical Care Medicine* (CCM), and *Critical Care* (CC) were the general critical care journals that had the four highest impact factors, provided by Thomson Reuters (New York, NY, USA). We thus selected these four journals. We selected two date ranges: pre-CONSORT from 2006 to 2007 and post-CONSORT from 2011 to 2012. We selected these periods because an interval of at least 24 months was considered necessary for dissemination of the reporting guidelines [16, 18], and previous similar studies in other fields have employed similar periods [16, 17]. The latest search was performed on 31 January 2015. Abstracts were included if they reported RCTs of any design. If the abstract of a potentially relevant article was unclear, we retrieved and assessed the full text to see if the study reported an RCT. We excluded abstracts of conference proceedings, observational studies, nonrandomized trials, quasi-RCTs, cost-effectiveness studies, diagnostic studies, letters, editorials, and reviews. We also excluded secondary and subgroup analyses of RCTs that had already been published.

### Assessment of abstracts

Two authors (A.K. and N.T.) independently reviewed abstracts and assessed each item of the CONSORT checklist [4]. We conducted a pilot review of 60 articles, and the uniformity of interpretation was thereby assured. Through this pilot review, we found that the setting of RCTs in the “Participants” section and the estimated effect size for “Outcomes” in the Results section were particularly underreported. This weakness was identified in a previous study [16], and we therefore conducted an additional assessment of these two items along with existing items in the CONSORT for abstract guideline. Occasionally, two items, such as “Registration” and “Funding,” were described on the journal websites, whereas they were not published in the PubMed record. We therefore preferentially assessed the descriptions on journal websites to overcome this discrepancy. For each item, we deemed reporting adequate in an abstract only when the abstract described the relevant item and fully adhered to the explanation and elaboration of CONSORT guideline for abstracts. We considered that an abstract was underreporting or inadequately reporting when a relevant item was either not reported or was not described as suggested by the explanation and elaboration of the guideline. Any disagreements were resolved by consensus decision, and when a decision was hard to make, agreement was reached through discussion with the third author (T.N.).

### Statistical analyses

For each item, we determined the number of abstracts that adhered to the CONSORT guideline for abstracts. Next, we determined the proportion of such abstracts among all included abstracts and subsequently determined proportions by journal. Pearson’s chi-square analysis was performed to compare adherence to the CONSORT guideline for abstracts between the pre-CONSORT and post-CONSORT periods for each item. Tests of statistical significance were two-sided, and values of *p* < 0.05 were considered significant. All analyses were conducted using Stata version 12.1 software (StataCorp, College Station, TX, USA).

## Results

**Fig. 1:**
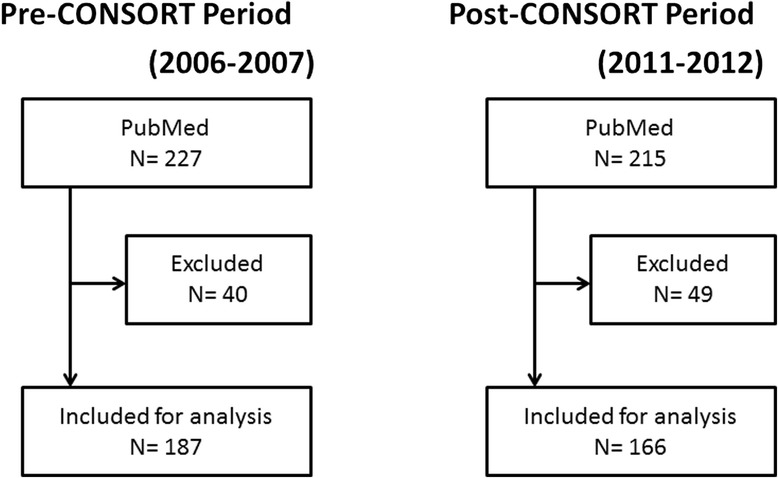
Flowchart of article selection. *CONSORT* Consolidated Standards of Reporting Trials

Our search yielded 227 and 215 abstracts from the pre-CONSORT and post-CONSORT periods, respectively (Table 1). After applying our inclusion and exclusion criteria, 185 and 166 abstracts in the pre-CONSORT and post-CONSORT periods, respectively, were analyzed (Fig. 1). These included 52 crossovers trials, 5 cluster-randomized trials, 2 factorial trials, and 3 N-of-1 trials; all others were parallel trials. Analyses by journal produced variable results with small numbers of abstracts; as a result, combined results are mainly shown (Table 2).

**Table 1:** The number of abstracts included in the analysis from both periods

Journal	Pre-CONSORT	Post-CONSORT	Total
AJRCCM	64 (35%)	45 (27%)	109 (31%)
CCM	66 (36%)	52 (31%)	118 (34%)
ICM	33 (18%)	34 (20%)	67 (19%)
CC	22 (12%)	35 (21%)	57 (16%)
Total	185 (100%)	166 (100%)	351 (100%)

*Abbreviations: AJRCCM American Journal of Respiratory and Critical Care Medicine*, *CC Critical Care*, *CCM Critical Care Medicine*, *CONSORT* Consolidated Standards of Reporting Trials, *ICM Intensive Care Medicine*

**Table 2:** Characteristics of included abstracts from both periods

	Pre-CONSORT	Post-CONSORT	*p* Value
Title	32.4	56.6	<0.001
Authors (for conference abstracts)	N/A	N/A	–
Trial design	22.7	16.3	0.130
Methods			
Participants	41.1	44.0	0.58
Setting^a^	41.1	44.0	0.58
Interventions	92.4	81.9	0.003
Objective	93.5	95.2	0.50
Outcome	31.4	49.4	0.001
Randomization	1.1	1.8	0.57
Blinding (masking)	3.2	4.2	0.63
Results			
Number randomized	41.6	37.4	0.41
Recruitment	2.7	4.2	0.44
Number analyzed	4.3	8.4	0.113
Outcome	4.9	16.9	<0.001
Effect size^a^	4.9	20.5	<0.001
Harms	11.9	27.7	<0.001
Conclusion	97.3	97.0	0.86
Trial registration	13.5	42.2	<0.001
Funding	1.6	13.9	<0.001

*CONSORT* Consolidated Standards of Reporting Trials, *N/A* not applicable

Data are percentages representing the proportion of abstracts that adhered to the CONSORT guideline for abstracts

^a^These items were additionally examined in this study

### Reporting of general items

Significantly more trials were described in the title as “randomized” in the post-CONSORT period (56.6%) than in the pre-CONSORT period (32.4%) (*p* < 0.01). A small proportion of trials from both periods described details of the trial design (pre-CONSORT 22.7%, post-CONSORT 16.3%).

### Reporting of trial methods

Participants were described in less than half of abstracts in both periods (pre-CONSORT 41.1%, post-CONSORT 44.0%), due to inadequate reporting of the setting. Interventions were adequately described in both periods, but a significant decrement in reporting of this item was noted in the post-CONSORT period (pre-CONSORT 92.4% vs post-CONSORT 81.9%; *p* < 0.01). Trials from both periods adequately described objectives or hypotheses of the trials (pre-CONSORT 93.5%, post-CONSORT 95.2%). Trials from both periods inadequately specified primary outcomes (pre-CONSORT 31.4%, post-CONSORT 49.4%), but a significant improvement was seen in the post-CONSORT period (*p* = 0.003). Randomization procedures and details regarding blinding were poorly reported in both periods, with no significant improvement in these items between periods.

### Reporting of trial results

Numbers of participants randomized in each group, trial status, and numbers of participants analyzed in each group were poorly reported in both periods, and no significant change was seen between periods. Primary outcomes for each group were inadequately reported in both periods, due to poor reporting of estimated effect sizes and precision. A significant increment in reporting of these items was noted in the post-CONSORT period (*p* < 0.001). Important adverse effects or side effects were also inadequately reported in both periods, although a significant improvement was seen in the post-CONSORT period (27.7%) (*p* < 0.001).

### Reporting of trial conclusions

Trial conclusions were sufficiently reported in both periods (pre-CONSORT 97.3%, post-CONSORT 97.0%).

### Reporting of trial registration and funding

Reporting of trial registration and funding was significantly improved in the post-CONSORT period compared with the pre-CONSORT period (*p* < 0.01). However, the frequency of adequate reporting of these items was still suboptimal in the post-CONSORT period (42.2% and 13.9%, respectively).

### Evaluations by journal

Noteworthy observations by journals are shown in Table 3. Trial designs were reported in less than 20% of abstracts across the journals, with CC being the lowest-reporting journal. Reporting of participants in the Methods section was adequate and improved over time in CCM but not in the other journals. Reporting of interventions in Methods sections declined in AJRCCM and ICM, and reporting of the numbers randomized in Results sections significantly declined in CC, significantly declined, between the two periods. Reporting of outcomes in Results sections significantly improved in AJRCCM and CCM. Reporting of trial registration in AJRCCM, CCM, and ICM significantly improved, with the increase in AJRCCM being the greatest. A tremendous increase in reporting of funding in AJRCCM was observed.

**Table 3:** Characteristics of included abstracts by individual journals

	AJRCCM			CCM			ICM			CC		
	Pre-CONSORT (%)	Post-CONSORT (%)	*p* Value	Pre-CONSORT (%)	Post-CONSORT (%)	*p* Value	Pre-CONSORT (%)	Post-CONSORT (%)	*p* Value	Pre-CONSORT (%)	Post-CONSORT (%)	*p* Value
Title	25.0	48.9	0.010	33.3	55.7	0.015	30.3	61.8	0.010	54.6	62.9	0.53
Authors (for conference abstracts)	–	–	–	–	–	–	–	–	–	–	–	–
Trial design	28.1	20.0	0.33	18.2	19.2	0.89	33.3	17.7	0.140	4.6	5.7	0.85
Methods												
Participants	10.9	17.8	0.31	68.2	92.3	0.001	66.7	29.4	0.002	9.1	20.0	0.27
Setting^a^	10.9	17.8	0.31	68.2	92.3	0.001	66.7	29.4	0.002	9.1	20.0	0.27
Interventions	92.2	77.8	0.032	92.4	90.4	0.69	93.9	67.7	0.007	90.9	88.6	0.78
Objective	95.3	97.8	0.50	89.4	96.2	0.170	97.0	94.1	0.57	95.5	91.4	0.56
Outcome	40.6	60.0	0.046	31.8	46.2	0.111	18.2	50.0	0.006	22.7	40.0	0.18
Randomization	0	0	–	0	3.9	0.108	0	0	–	9.1	2.9	0.31
Blinding (masking)	0	2.2	0.23	6.1	9.6	0.47	6.1	0	0.145	0	2.7	0.42
Results												
Numbers randomized	35.9	33.3	0.78	39.4	48.1	0.35	39.4	29.4	0.39	68.2	34.2	0.013
Recruitment	6.3	8.9	0.60	1.5	0	0.37	0	8.8	0.081	0	0	–
Numbers analyzed	4.7	6.7	0.66	4.6	7.7	0.47	3.0	11.8	0.174	4.6	8.6	0.56
Outcome	7.8	22.2	0.032	3.0	19.2	0.004	3.0	11.8	0.174	4.6	11.4	0.37
Effect size^a^	7.8	31.1	0.002	3.0	21.2	0.002	3.0	14.7	0.094	4.6	11.4	0.37
Harms	20.3	24.4	0.61	12.1	30.8	0.012	0	29.4	0.001	4.6	25.7	0.041
Conclusion	98.4	95.6	0.37	98.5	100	0.37	90.9	94.1	0.62	100	97.1	0.42
Trial registration	18.8	62.2	<0.001	0	15.4	0.001	0	17.7	0.011	59.1	80.0	0.087
Funding	0	48.9	<0.001	0	0	–	9.1	0	0.072	0	2.9	0.42

*Abbreviations: AJRCCM American Journal of Respiratory and Critical Care Medicine*, *CC Critical Care*, *CCM Critical Care Medicine*, *CONSORT* Consolidated Standards of Reporting Trials, *ICM Intensive Care Medicine*

Percentages represent the proportion of abstracts that adhered to the CONSORT guideline for abstracts

^a^These items were additionally examined in this study

## Discussion

The findings of our study suggest that, compared with the pre-CONSORT period, reporting of some items (title, primary outcomes in both Methods and Results, interventions, harms, trial registration, funding) in abstracts of critical care trials was improved in the post-CONSORT period. We examined 18 items in the main analysis; when the significance threshold was adjusted with the Bonferroni correction (*p* < 0.003), significant improvement in reporting the same items (except interventions) was still noted. However, even in the post-CONSORT period, more than 50% of trials inadequately reported trial design, participants, and outcomes in the Methods section; randomization procedures, details of blinding, numbers of participants randomized to each group, trial status, numbers of participants analyzed in each group, and outcomes in the Results section; and adverse effects, trial registrations, and funding. This tendency was generally observed across the four journals, although there were some small exceptions and variations. Overall adherence to the CONSORT for abstract guideline was thus considered suboptimal.

Our results were broadly similar to those of previous studies [15–17]. Can et al. assessed abstracts of trials published in four leading anesthesia journals and found that the quality of reporting abstracts was improved in the post-CONSORT period (2008 to 2009) compared with the pre-CONSORT period (2006 to 2007). Adherence to the CONSORT for abstract guideline was only 35.2% even in the post-CONSORT period [15]. Ghimire et al. investigated whether the overall quality of oncology trial abstracts was improved in the post-CONSORT period (2010 to 2012) compared with the pre-CONSORT period (2005 to 2007), and they found that adherence to the CONSORT for abstract guideline remained suboptimal (55.2%) [14]. Fleming et al. suggested that the reporting quality of trial abstracts published in leading orthodontic journals did not change significantly between the two periods of 2006–2008 and 2009–2011 [19]. Mbuagbaw et al. examined 100 randomly selected trials published in each of 2007 and 2012 in six leading general medicine journals (*New England Journal of Medicine*, *Lancet*, *BMJ*, *JAMA*, *Annals of Internal Medicine*, *CMAJ*) [17]. Abstracts in these journals tended to report the title (66%), trial design (59%), and Methods section except randomization and blinding (about 60% or more), whereas in 2007 they tended to underreport numbers randomized (53%) and analyzed (26%), recruitment (25%), harms (53%) in the Results section, Conclusions section (22%), and funding (0%). In 2012, in contrast, most items were more frequently reported than in 2007, but randomization (13%) and blinding (59%) in the Methods section, numbers randomized (61%), recruitment (49%), harms (60%) in the Results section, and Conclusions section (26%) remained lower than the other items. Significant improvements were seen in the title, trial design, participants, objectives, randomization, and blinding in the Methods section, as well as recruitment and numbers analyzed in the Results section and funding (*p* < 0.05). Overall, all items except interventions and objectives in the Methods and Conclusions sections were much less frequently reported in critical care journals than in leading general medicine journals. In contrast to general medicine journals, significant improvement was observed in different items, except the title and funding in critical care trial abstracts. Hays et al. examined RCT abstracts published in five leading general medical journals (*New England Journal of Medicine*, *Lancet*, *BMJ*, *JAMA*, *Annals of Internal Medicine*) from 2011 to 2014 and suggested that there was still low adherence to the guideline (overall adherence 67%) [20].

The proportion of adequate reporting of interventions declined significantly between the two periods in our study (pre-CONSORT 92.4%, post-CONSORT 81.9%). A similar trend was also observed in two previous studies, with varying significance [15, 17]. This might have been due to declines in some, if not all, journals, but reasons were not possible to elucidate in any of these cases.

Previous studies have also suggested that trials published after the announcement of the CONSORT for abstract guideline tended to inadequately report title, trial design, and primary outcomes in Methods sections, randomization procedures, details of blinding, numbers of patients randomized and analyzed in each group, trial status, and primary outcomes in Results sections, trial registration, and funding [5, 7–10, 14–17, 19]. These tendencies were confirmed in our study.

There are several hypotheses regarding the suboptimal adherence of critical care journals to the guideline. One is that the authors were unaware of the CONSORT guideline for abstracts in the first place. They might prefer to concentrate their efforts on the manuscripts, or the adherence to the guideline for abstracts could be felt to be extra “work” for the authors [21]. Specifically regarding the harms, critical care trials often examine undesirable outcomes such as mortality, and harms could be omitted from the abstracts [17]. Also, when the intervention of interest was found be effective, it is imaginable that the harms were not discussed as well.

One potential argument is that these abstracts underreported important information owing to the space constraints set by journals. Currently, none of the four journals we selected endorse the CONSORT guideline for abstracts. The word limits for each journal were as follows: AJRCCM, 250 words; CCM, 300 words; ICM, 250 words; and CC, 350 words. However, the explanation and elaboration of the CONSORT guideline for abstracts considered that items in the checklist could be incorporated within a limit of 250–300 words [4]. Hopewell et al. suggested that implementing the CONSORT guideline for abstracts led to improvements in the reporting of trial abstracts in their interrupted time-series analysis of major general medicine journals [22]. Endorsing the CONSORT checklist as a journal policy is also known to be associated with improved reporting of trials [23]. Thus, first of all, it is desirable for editors of critical care journals to consider adopting the CONSORT guideline for abstracts as a journal editorial policy as a prerequisite of submission. This would facilitate adoption of the CONSORT guideline for abstracts by authors of trial manuscripts, making abstracts sound and readable. Likewise for reviewers, completeness and efficiency of assessing abstracts could be improved if the CONSORT guideline for abstracts were better disseminated.

Our study has some strengths. We included a large number of critical care trial abstracts. Two reviewers independently evaluated each item of the checklist proposed by the CONSORT guideline for abstracts in a standardized manner. This represents one of few studies to assess adherence to the CONSORT guideline for abstracts, along with changes in the quality of reporting for trial abstracts between two periods in any domain of medicine.

On the other hand, some limitations must be considered. First, we focused on only four leading journals, which may well have been unrepresentative of the totality of critical care medicine journals. Currently, 27 journals are listed in the field of critical care medicine by Thomson Reuters, and the four journals we selected are ranked among the top five according to impact factor. A previous study suggested that lower impact factors were associated with lower quality of reporting abstracts [16]. The reporting quality of abstracts in other journals would thus be expected to be lower than our findings, meriting further assessment of other critical care journals. Second, a few years have passed since the second study period in our investigation. The trial abstracts published in five leading general medical journals from 2011 to 2014 still showed low adherence to the guideline (overall adherence 67%) [20], and thus an update for each area is warranted. To our knowledge, the present study is the first one to examine adherence to the CONSORT guideline for abstracts in critical care journals. This study thus should serve as the foundational investigation for the update on the same topic in critical care medicine and other areas. Third, no clear consensus or validated methods were seen regarding the assessment of quality for trial abstracts. Previous studies have assessed abstracts by each item of the CONSORT guideline for abstracts [7, 8, 12, 14, 15], measured overall quality by counting the number of items reported adequately [16, 17], or used their own criteria [9, 10, 19]. However, whether each item can be weighted equally is questionable. Even so, these studies consistently reported similar results and inadequately reported items, supporting our methods of assessment.

## Conclusions

Our findings suggest that the reporting quality of abstracts in critical care journals has improved since the announcement of the CONSORT guideline for abstracts. However, overall adherence to the guideline remains suboptimal. Given the lack of editorial policy to implement the guideline in critical care journals, journal editors need to require authors to follow the guideline to promote further improvements in the quality of reporting for abstracts and subsequent readability.

## Abbreviations

AJRCCM: *American Journal of Respiratory and Critical Care Medicine*
CC: *Critical Care*
CCM: *Critical Care Medicine*
CONSORT: Consolidated Standards of Reporting Trials
ICM: *Intensive Care Medicine*
RCT: Randomized controlled trial
